# Do group ensemble statistics bias visual working memory for individual items? A registered replication of Brady and Alvarez (2011)

**DOI:** 10.3758/s13414-020-02209-6

**Published:** 2020-12-02

**Authors:** Frank Papenmeier, J. David Timm

**Affiliations:** Department of Psychology, University of Tübingen, Schleichstr. 4, 72076 Tübingen, Germany

**Keywords:** Ensemble perception, Summary statistics, Registered replication, Bayes Factor Design Analysis, Amazon Mechanical Turk

## Abstract

We performed a registered and precise replication of Experiment 1 reported in Brady and Alvarez (*Psychological Science*, *22*, 384–392, 2011). The original experiment found that participants, who were asked to memorize the size of differently colored circles, reported the size of a probed circle biased toward the mean size of the same-colored group. Because our previous three unpublished replication attempts failed to find this effect, we powered the present registered replication using a Bayes Factor Design Analysis such that it provided compelling evidence regarding the presence or absence of the reported bias with a high probability, even under the assumption of smaller effect sizes. Thus, we recruited 663 participants through Amazon Mechanical Turk. We observed both a significant bias and strong Bayesian evidence in favor of the existence of a bias over the null hypothesis. Thus, our results can be considered a successful replication of the original findings, although with a considerably smaller effect size. We discuss the role of data quality when recruiting participants with Amazon Mechanical Turk. The present findings corroborate the idea that memory representations of individual objects are influenced by summary statistics.

## Introduction

Whereas it is well established that individuals can hold only a limited amount of information in visual working memory, there is still a debate on the nature of the retained information and its structure. Besides the massive amount of research focusing on the number and precision of the represented units (e.g., Bays et al., 2009; Luck & Vogel, 1997; Wheeler & Treisman, 2002; Zhang & Luck, 2008), there is also an increasing amount of research suggesting that visual working memory is structured in a hierarchical manner with the representation of individual items being influenced by higher layers representing ensemble statistics of the retained objects (e.g., Brady et al., 2011; Brady & Tenenbaum, 2013; Orhan & Jacobs, 2013). Thus, memory for individual items is influenced by the other retained items introducing systematic biases regarding a number of features, such as size (Brady & Alvarez, 2011), color (Nassar et al., 2018), or spatial location (Lew & Vul, 2015; Orhan & Jacobs, 2013).

**Fig. 1:**
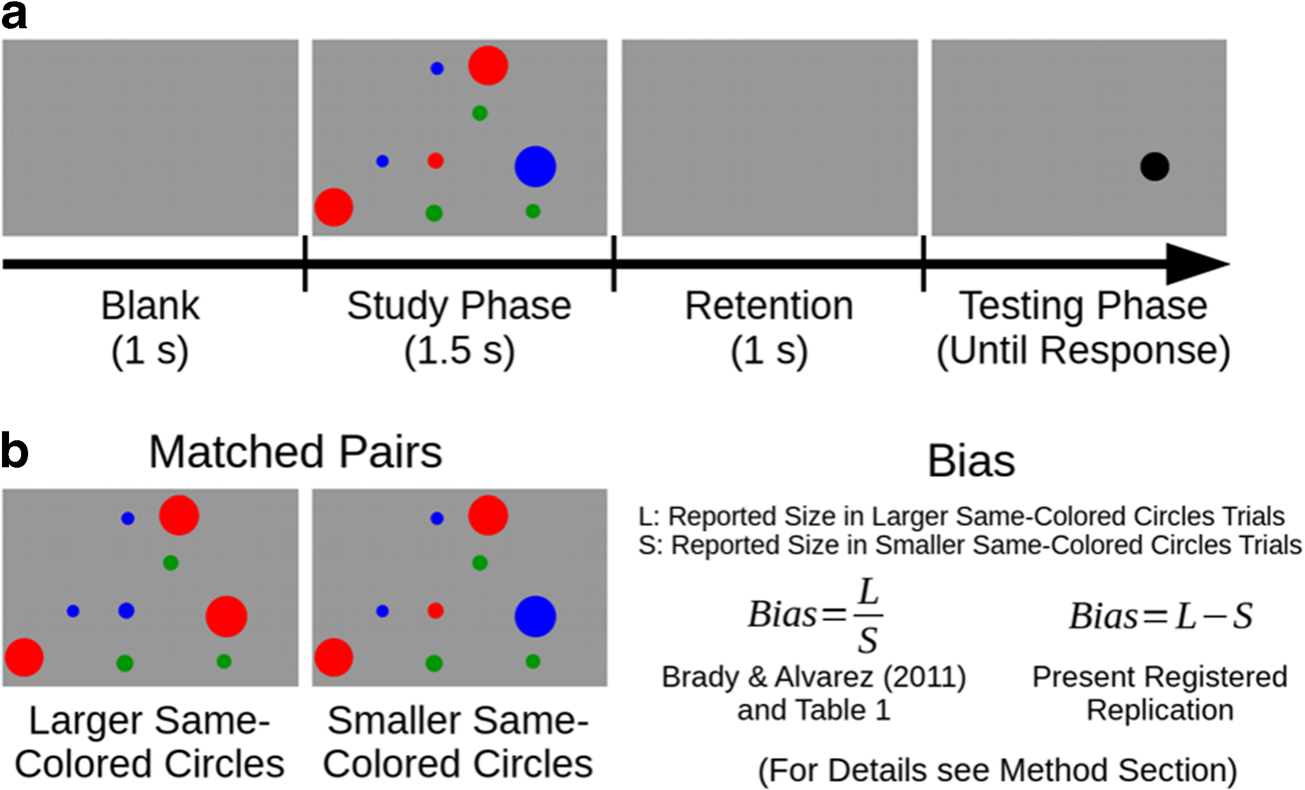
(**A**) Participants memorized the size of the red and blue circles while ignoring the green circles. During the testing phase, participants adjusted the size of a black probe circle to match the size of the originally encoded object at that location as close as possible. (**B**) The experiment consisted of 15 matched pairs (30 trials). For each matched pair, the color of the probed circle and a circle of the other color (red/blue) was swapped such that the probed circle was presented either in the context of larger same-colored circles or smaller same-colored circles. The dependent measure *bias* was calculated based on the reported size of the probed circle when presented in each context. Please refer to the Methods section for further details on the different formula applied for the calculation of bias

One influential study in this field presented participants with displays containing nine circles (three red, three blue, three green) and asked them to memorize the size of the red and blue circles but to ignore the green circles (Experiment 1 reported in Brady & Alvarez, 2011; see Fig. 1 for an illustration of the task). They found that the participants’ memory of the size of an individual object was biased toward the mean group-size of simultaneously retained same-colored objects. This finding is particularly exciting because it has two implications for the representation of information in visual working memory. First, items in visual working memory are represented not only individually but also as a group with group information affecting the representation of individual items. This implication is supported by a number of related findings, such as ratings on face expressions or face attractiveness being biased by the surrounding group of faces (Corbin & Crawford, 2018; Griffiths et al., 2018; Walker & Vul, 2014), reported object orientations being biased toward the group mean (Utochkin & Brady, 2020; but see also Huang, in press, for the role of strategic guesses on such memory biases), or the averaging of the spatial frequencies of two memorized Gabor patches (Dubé et al., 2014). A second implication that we can derive from the study by Brady and Alvarez (2011) is that visual working memory is capable of representing not only a single overall ensemble statistic, such as the mean size of all retained objects, but also multiple (at least two) separate ensemble statistics, such as the mean size of two differently colored groups of objects. However, evidence supporting this second implication is sparse. Despite the original finding being highly consistent across participants, recent research demonstrated that the strength of this bias is influenced by individual differences in sensory processing (Lowe et al., 2016) and the individual’s level of autistic traits (Lowe et al., 2018). In an attempt to further corroborate this second implication, we performed three unpublished replication attempts that – to our surprise – did not show this bias. In the following, we briefly report those unpublished studies.

**Fig. 2:**
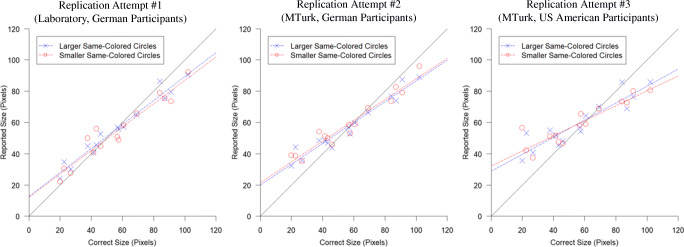
Reported size of the probed object (averaged across participants) such that each dot represents one of the 30 trials (15 matched pairs, once with larger same-colored circles and once with smaller same-colored circles, see Methods section for details) similar to Fig. 3a shown in Brady and Alvarez (2011). In contrast to Brady and Alvarez (2011), we did not observe a systematic shift of the probed object being reported as larger when presented in the larger same-colored circles context than when presented in the smaller same-colored circles context

**Fig. 3:**
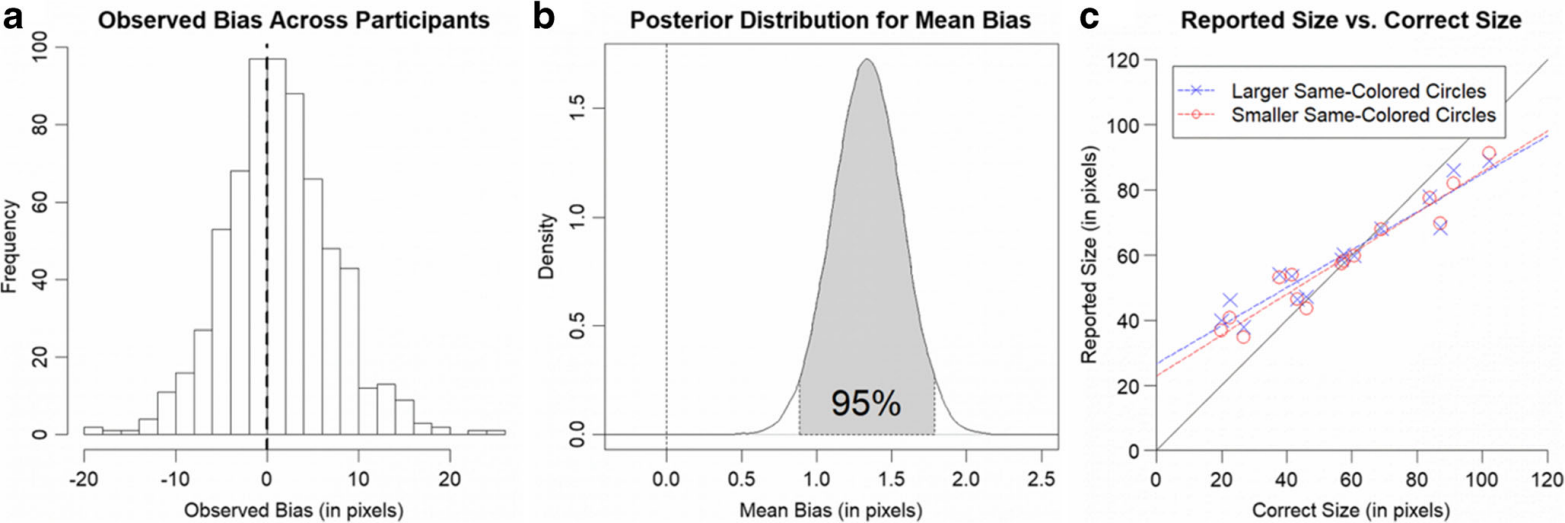
(**A**) Histogram of observed biases across participants. (**B**) Density plot of posterior distribution for mean bias (using 100,000 sampling iterations). (**C**) Depiction of reported size of the probed object (averaged across participants) for all trials (15 matched pairs) enabling an exploratory visual comparison of results with our previous replication attempts (see Fig. 2) and the original experiment (see Fig. 3a in Brady & Alvarez, 2011)

We conducted three unpublished studies that were designed to replicate the procedure and analysis of the first experiment reported in Brady and Alvarez (2011) as close as possible.^1^ Across our three replication attempts,^2^ we increasingly matched the experimental setting used within the original experiment (US American participants recruited through Amazon Mechanical Turk). A summary of the replication attempts is given in Table 1. Whereas the two-sided *t*-test comparing the obtained bias against a ratio of 1.0 was significant in the original experiment, *p* < .001, we did not observe significant effects in our experiments, all *p*s ≥ .110. In order to enable a visual comparison between the data obtained in our replication attempts and the original study, we created an illustration with our data (see Fig. 2) that resembles Fig. 3a shown in Brady and Alvarez (2011). As is evident from this figure, the size of the probed objects across participants was not systematically biased by whether the same-colored objects were larger or smaller in our experiments.

**Table 1:** Summary of bias and statistics found in the original Experiment 1 reported by Brady and Alvarez (2011) and our previous three replication attempts

Study	Experimental Setting	N	Bias, Mean (SD)	p-value	BF_10_	Cohen’s d_z_
Experiment 1 in Brady & Alvarez (2011)	Online (Mechanical Turk) US American Participants	21	1.11 (0.12)	< .001	69.15	0.91
Replication Attempt #1	Laboratory German Participants	21	1.03 (0.09)	.110	0.75	0.36
Replication Attempt #2	Online (Mechanical Turk) German Participants	21	0.98 (0.09)	.404	0.31	-0.19
Replication Attempt #3	Online (Mechanical Turk) US American Participants	21	1.01 (0.13)	.821	0.23	0.05

*Note.* Bias values reported in this table were calculated following the procedure used by Brady & Alvarez (2011), i.e., the “ratio of averages” approach (see Methods section for details). The reported Bayes Factors were calculated with a Cauchy prior with a scale of sqrt(2)/2

One limitation of both the original study and our replication attempts is the rather low sample size of 21 participants per study. Thus, with the present paper, we performed a registered and precise replication of Experiment 1 reported in Brady and Alvarez (2011). We did so by extending the original analysis with a Bayesian approach and recruiting a sample size large enough to gain compelling evidence regarding the presence or absence of the reported bias with a high probability, even under the assumption of smaller effect sizes.

## Registered replication

This experiment is a precise replication of Experiment 1 reported in Brady and Alvarez (2011). Thereby, we focused on the main finding of the original experiment, namely, that the reported size of a probed object was biased toward the mean size of the same-colored group. Importantly, our previous replication attempts indicated that this bias might either be smaller than previously assumed or the previous rejection of the null hypothesis might have occurred due to a type 1 error. Thus, it was important to power this replication not only for the alternative hypothesis, but also to quantify the evidence in favor of the null hypothesis and power this replication accordingly. Therefore, we extended the original analysis by a Bayesian approach. In particular, we ran a Bayesian *t*-test in order to determine the Bayes Factor BF_10_. We used a Cauchy prior with a scale of sqrt(2)/2.

### Method

#### Power analysis and Bayes Factor Design Analysis

We targeted for a sample size that was large enough to also detect small effect sizes of 0.3 (Cohen’s *d*_*z*_) with a power of .80. Therefore, we first conducted a power analysis using G*Power (Faul et al., 2007). This revealed a required sample size of 90 participants to detect an effect of 0.3 with a two-sided *t*-test against a constant at an alpha level of .05.

Because the aim of this replication was to gain compelling evidence for either the alternative hypothesis or the null hypothesis, we then conducted a Bayes Factor Design Analysis for a fixed-n design (Schönbrodt & Wagenmakers, 2018). Therefore, we ran two Monte Carlo simulations (10,000 repetitions per sample size and simulation; R code available at https://osf.io/ha45r/ ), one under the assumption of a true effect size of 0.3 in order to determine the required sample size to gain a Bayes Factor BF_10_ ≥ 10 (true-positive evidence) with a probability of at least .80 and a second simulation under the assumption of no effect in order to determine the required sample size to gain a Bayes Factor BF_10_ ≤ 0.1 (true-negative evidence) with a probability of at least .80. Whereas the first simulation resulted in a required sample size of 180 participants, the second simulation resulted in a required sample size of 663 participants.

Based on these considerations, we decided to recruit 663 participants. At this sample size, the true power of the two-sided *t*-test under the assumption of an effect size of 0.3 was larger than .999. Further, when considering Bayes Factor boundaries of 0.1 and 10, this sample size left us with a probability of > .999 for true-positive evidence, < .001 for false-negative evidence, and < .001 for inconclusive evidence under the assumption of a true effect of 0.3 and a probability of .802 for true-negative evidence, < .001 for false-positive evidence, and .197 for inconclusive evidence under the assumption of no effect. Please note that under those assumptions, there was only a very low probability of concluding that a true effect exists despite no true effect being present or vice versa.

#### Participants

We recruited 663 participants (342 female, 321 male, 0 intersex; *M*_age_ = 43.45 years, *SD*_age_ = 12.76 years, age range 18–91 years) using Amazon Mechanical Turk. All participants gave informed consent and were paid $1.15. Further, participants were required to be located in the USA, not to have participated in our previous replication attempt, to have at least 5,000 approved HITs, and to have a HIT approval rate greater than 98%.^3^

We excluded all participants from the data set and replaced them with new participants if they did not provide complete data sets (e.g., aborted experiment early, *N* = 20), reported not wearing glasses or contact lenses despite needing them (*N* = 9), reported being aware of a color vision impairment (*N* = 19), reported not being able to view the contents of the experiment in its entirety (*N* = 2), did not select “elephant” as the largest animal from a list of animals (*N* = 0), or responded without prior adjustment of the size of the probe circle in more than two trials (*N* = 25). Further, if a participant completed the experiment multiple times, we included only the first participation into the data set and removed and replaced all subsequent participations (*N* = 7).

#### Stimuli

We collected demographic information, self-reports on vision impairments and the visibility of the experimental stimuli, as well as a question intended to ensure that the participants were English speaking and read the instructions (select largest animal from list of animals) on Mechanical Turk. The main experiment was programmed with PsychoPy Builder (Peirce et al., 2019) and exported and run as an PsychoJS experiment on Pavlovia (https://pavlovia.org/). The experiment is available as open material at https://osf.io/ha45r/. We replicated the stimuli used by Brady and Alvarez (2011) in their Experiment 1 as precisely as possible.^4^ Thus, within each trial, we presented nine circles (three red, three blue, three green) on a gray background measuring 600 × 400 pixels.^5^ We presented the 15 matched pairs (30 trials) from the original study. Thus, circle locations, colors, and sizes matched the original study. Further, we also added a random jitter of ±10 pixels to each circle location for each participant individually to prevent collinearities (with the restriction that objects were not allowed to overlap or extend beyond the background after jitter was applied). We informed the participants that a keyboard and a mouse were required in order to complete the task and that the task ran only with current versions of either the Firefox or the Chrome browser. As in the original study, the size and resolution of the monitor was not controlled. However, the participants could only start the experiment if the experimental task fit within the browser’s drawing area. We also added a question to the Mechanical Turk questionnaire about whether the participants could view all content in its entirety.

#### Procedure

At the beginning of the experiment, each participant filled in a form on Mechanical Turk giving information on the age, sex, and potential vision impairments. They were informed about data confidentiality and that the recorded data would be published in an anonymized manner. They then clicked on a link that took them to the main experimental task that was presented using Pavlovia. They were instructed about the trial procedure (1.5-s study phase, 1-s retention, testing phase until response) and to remember the size of the red and blue circles but to ignore the green circles. This instruction resembled the information provided in the article by Brady and Alvarez (2011) but was not an exact copy of the original instruction as this was not available to us. After reading a statement on data confidentiality and the voluntary nature of participation, they started the experimental task. Each participant saw the trials in an individual random order with the restriction that matched displays could not follow directly one after the other. For each trial, there was a 1-s blank before the study display was shown for 1.5 s. The study display was followed by a blank retention phase of 1 s. Finally, a single black circle with a random initial diameter between 15 and 95 pixels appeared at the original location of the probed red or blue circle. The participants then moved the mouse upwards and downwards to adjust the size of this circle to match the size of the originally encoded object at that location as close as possible. They locked in their responses by clicking a mouse button and then proceeded to the next trial. If the participants clicked a mouse button without performing the task (i.e., did not move the mouse to adjust the size of the probe circle), a reminder about moving the mouse for diameter adjustment was displayed, and they could only advance to the next trial once they did so.

#### Measuring bias: Averaging ratios versus ratio of averages versus difference score?

Taking advantage of the fact that the probed circle had the same actual size in both trials of each matched pair, Brady and Alvarez (2011) calculated bias as the ratio of the reported size of the probed circle when presented in the trials containing the larger same-colored group divided by the reported size of the probed circle when presented in the trials containing the smaller same-colored group (see Fig. 1). Thus, a bias of responses toward the mean size of the same-colored circles should result in a bias larger than 1.0, whereas a ratio of 1.0 would indicate that responses were not biased. Importantly, there are two versions of determining the bias on the participant level, namely, calculating the ratio of each trial and averaging across ratios (*averaging ratios*) or calculating the average of the reported size for the larger same-colored group trials and smaller same-colored group trials for each participant and then computing the ratio of those averages (*ratio of averages*).

We noted that the two versions of calculating bias provided quite different results when applied to the data of our previous replication attempts, namely, resulting in a significant bias when calculated using the averaging ratios approach for each replication attempt but resulting in a non-significant bias when calculated using the ratio of averages approach for each replication attempt. Given this observation and considering that calculating the arithmetic mean across ratios, which are open ended towards the upper bound but limited towards zero, might cause statistical artifacts potentially leading to largely inflated alpha errors when analyzed with a *t*-test, we ran a simulation investigating this concern. For this simulation, we applied the following procedure (R code available online at https://osf.io/ha45r/): (1) simulate data for the 15 matched pairs as used in the present experiment for a number of participants under the assumption of the null, that is, no difference in reported size between trials with a larger same-colored group and trials with a smaller same-colored group; (2) run a *t*-test and obtain the p-value; (3) repeat steps one and two 100,000 times and determine the observed alpha error, that is, the proportion of trials with significant *t*-tests (against an intended alpha of .05). Given a sample size of 21 participants as in the original experiment, this revealed a massively inflated alpha error of .998 for the averaging ratios approach and a only mildly inflated alpha error of .053 for the ratio of averages approach. However, under the assumption of a larger sample size, such as the 663 participants that we had planned for the present registered replication, both approaches resulted in inflated alpha errors, with 1.0 for the averaging ratios approach and .387 for the ratio of averages approach. Whereas the Brady and Alvarez (2011) article did not specify which of the two approaches they had used for their original analysis, the analysis scripts provided to us by Tim Brady showed that they had used the ratio of averages approach, which performed with a reasonable alpha error in our simulation, at least under the assumption of a sample size of 21 participants. Thus, our failure to replicate the original finding with our previous replication attempts cannot be attributed to differences in the way the ratios were averaged. However, it seems that the replications reported in Lowe et al. (2016, 2018) calculated bias using the average of ratios approach rendering their significant overall bias effects meaningless and raising the question of what their results might have been if a different measure for bias had been used.

Because our present registered report targeted a large sample size in which the ratio of averages approach also results in a considerably inflated alpha error, we calculated bias not as a ratio but as a difference score for this registered report; that is, we defined bias as the reported size of the probed circle when presented in the trials containing the larger same-colored group minus the reported size of the probed circle when presented in the trials containing the smaller same-colored group (see Fig. 1). Thus, a bias of responses toward the mean size of the same-colored circles should result in a bias larger than zero, whereas a bias of zero would indicate that responses were not biased. For this difference score measure, first averaging and then calculating the difference score or first computing the difference score and then averaging does result in equivalent results. Further, running the above simulation to determine alpha errors for the difference score measure confirmed that alpha errors were not inflated with an observed alpha error of .050 for a sample size of 21 participants and an observed alpha error of .051 for a sample size of 663 participants. Finally, we performed a visual inspection of the distribution of the difference scores for each matched pair using violin plots based on the data of our previous three replication attempts. We did not observe systematic variations in the distributions with increasing circle size, indicating that calculating the mean difference scores across matched pairs should weight matched pairs with small and large probed circles similarly.

#### Pre-planned analysis

In order to provide a precise replication of the analysis plan used by the original study, we first assessed whether participants could perform the size memory task by comparing actual performance with the empirical measure of chance performance introduced by Brady and Alvarez (2011). Thus, we first randomly paired each participant’s responses with the correct answers from different trials. We then calculated the mean absolute error in reported size for the actual trials and the randomly paired trials, and then compared those error values across participants with a two-sided paired *t*-test.

Next, we replicated the analysis for the main finding of the original experiment, namely, that the reported size of a probed circle was biased toward the mean size of the same-colored group. Thus, for each participant, we determined bias as a difference score as described above. We then compared bias across participants against the constant of 0 using a two-sided *t*-test and calculated the effect size Cohen’s *d*_*z*_.

In a next step, we ran a two-sided Bayesian *t*-test on bias against the constant of 0 using the R-package BayesFactor (Morey & Rouder, 2018) in order to determine the Bayes Factor BF_10_. This was done in order to quantify the evidence in favor of the alternative evidence that the mean bias deviates from 0 over the null hypothesis. Further, we reported the posterior distribution and 95% credible interval for mean bias (using 100,000 sampling iterations) in order to gain an impression of the size of the bias effect.

In a final step, we re-ran both analyses (*t*-test and Bayesian *t*-test) including only those participants who showed a mean absolute error lower than 25 pixels. This was done as a sanity check to ensure that the results on ensemble perception hold also when only including those participants having performed fairly well in the memory task overall.

### Results

The participants performed well in the size memory task overall, with participants’ mean absolute error in reported size (*M* = 16.69 pixels, *SE* = 0.29 pixels) being lower than chance (*M* = 30.90 pixels, *SE* = 0.19 pixels), *t*(662) = -46.39, *p* < .001, *d*_*z*_ = -1.80, 95% CI [-1.92, -1.68], and similar to the value reported in the original study (*M* = 16.4 pixels, *SE* = 1.7 pixels). Importantly, we observed a bias (*M* = 1.34 pixels, *SE* = 0.23 pixels; see Fig. 3A) deviating significantly from zero, *t*(662) = 5.83, *p* < .001, *d*_*z*_ = 0.23, 95% CI [0.15, 0.30]. That is, based on this analysis, we replicated the main finding of the original experiment, namely, that the reported size of the probed circle was biased toward the mean size of the same-colored group. The observed effect size was, however, much smaller than in the original study. Further, in contrast to 90.5% of the participants (19 out of 21) showing a bias in the expected direction in the original study, we observed a bias larger than zero for only 57.8% of the participants (383 of 663).

The Bayesian *t*-test also provided strong evidence in favor of the alternative hypothesis over the null hypothesis, BF_10_ = 634953. That is, based on the present data set there is strong evidence in favor of participants showing a bias of reporting the size of the probed object toward the mean size of the same-colored group. Despite the strong evidence for the alternative hypothesis that mean bias deviates from zero, the posterior distribution (see Fig. 3B) and the 95% credible interval for mean bias [0.88 pixels, 1.79 pixels] indicate that the size of this bias effect is rather small in pixels units.

As a sanity check of our data, we re-ran the *t*-tests and Bayesian *t*-test including only those participants who showed a mean absolute error lower than 25 pixels (572 participants, 86.27% of the data). We again observed that bias (*M* = 1.58 pixels, *SE* = 0.25 pixels) deviated from zero significantly, *t*(571) = 6.40, *p* < .001, *d*_*z*_ = 0.27, 95% CI [0.18, 0.35], BF_10_ = 16599137.

### Discussion

Previous research suggests that the memory for individual items is systematically biased by the other items retained in memory (e.g., Brady et al., 2011; Brady & Alvarez, 2011; Brady & Tenenbaum, 2013; Lew & Vul, 2015; Nassar et al., 2018; Orhan & Jacobs, 2013). With the present registered report, we performed a precise replication of one of these findings, namely, that the reported size of colored discs is biased toward the mean size of a same-colored group (Brady & Alvarez, 2011). Given the failure to replicate this finding within three of our unpublished studies, we employed a Bayesian approach in this registered replication and powered this experiment to gain compelling evidence for either the alternative hypothesis (bias exists) or the null hypothesis (no bias). The obtained results provide strong evidence in favor of the alternative hypothesis over the null hypothesis. Thus, our results replicate the main finding of Brady and Alvarez (2011), although with a smaller effect size.

Whereas in the original experiment there was a consistent result pattern with almost all participants showing the bias, the observed bias was much more diverse in our present experiment. Given the low number of trials in both experiments, however, we cannot draw strong conclusions from this observation. Besides an indication for individual differences in the processing of ensemble statistics across participants, this might simply reflect a large measurement error that might decrease if one uses more measurements per participants. Nonetheless, previous results employing the same task (Lowe et al., 2016, 2018) indicate that there might indeed be differences in the processing of summary statistics across individuals. Further progress along this line of research might help to gain insights into the processes underlying the influence of summary statistics on memory representations.

Although the effect size observed in our registered replication is substantially lower than the effect size reported in the original experiment, it is just somewhat lower than in our first unpublished lab study reported in Table 1 (see replication attempt #1). Therefore, it seems that data collected through online recruitment systems such as Amazon Mechanical Turk can lead to result patterns similar to data collected within the lab. There is one important exception, however: This only applies when employing strict requirements for participation, such as at least 5,000 approved HITs with an approval rate greater than 98% as used in the data set reported above. As mentioned in Footnote 3, we performed a first round of data collection without any requirements other than participants being located in the USA (as in the original study published in 2011). Following our registered methods, this resulted in a high rate of participants that had to be replaced and less than one-half of the final data set fulfilling our registered 25 pixels criterion for the sanity check. Given the poor data quality, we re-ran data collection by applying the above restrictions as suggested by an MTurk blog post (Amazon Mechanical Turk, 2019), eventually resulting in much better data quality. Therefore, it seems likely that the pool of participants accessible through Amazon Mechanical Turk might have changed drastically within the 10 years lying between the original study and our registered replication. This is something that should be taken into consideration when trying to replicate other studies that recruited participants online.

Our successful replication of the main finding of Brady and Alvarez (2011) provides further evidence that memory representations of individual objects are influenced by summary statistics. In particular, the reported size of colored discs is biased toward the mean size of a same-colored group corroborating the general idea that the memory representation of individual objects is influenced by summary statistics. Regarding the specific experimental design employed in our registered replication, however, the effect size seems to be smaller than previously assumed. Thus, future research employing this task should employ improved versions of the design, such as applying a more reasonable number of trials per participant in order to reduce measurement error. In addition, we propose conducting further research investigating both the mechanism underlying the influence of summary statistics on memory representations and the boundary conditions and prerequisites of their emergence.

## Footnotes

1 All experiments were conducted using PsychoJS and Pavlovia similar to the registered replication reported in the present paper. However, due to an error in the session files, the black probe circle used to report object size was minimally misaligned from the originally encoded circle for a total of four trials across three participants (range 1–6.7 pixels). This error did not affect the actual size of the black probe circle nor the recorded response. Removing the affected trials and respective matched trials from the analysis did not change the pattern of reported results. Note that we used new session files without this error for the data collection of the registered replication reported in the present paper.

2 Please note that Brady and Alvarez (2011) report multiple successful replications of their Experiment 1 within their Online Supplementary Material. However, because those replications used either different sets of displays or combined their Experiment 1 with their Experiment 2A to a within-participants experiment, thus also changing the original procedure, we did not include them in Table 1 and in the presentation of our previous replication attempts.

3 The two requirements for approved HITs and approval rate were defined only after the collection of a first data set due to poor data quality. Without those requirements, after removing and replacing 1,024 participants of the sample based on our registered replacement criteria, we stopped data collection at a sample size of 598 participants. The analysis of those 598 participants then revealed that only 48.7% of these data set fulfilled the 25 pixels criterion defined as a sanity check in our pre-planned analysis. Thus, we then decided to define the two additional requirements on approved HITs and approval rate, following the recommendations made in a MTurk blog post (Amazon Mechanical Turk, 2019), and decided to recollect the data for this registered report. Prior to this recollection, we also optimized the presentation of questions in the MTurk questionnaire. Note that we also made available the first data set together with screenshots of the first MTurk questionnaire on Open Science Framework.

4 We thank Tim Brady for sharing the data and analysis script including details on the stimulus displays of the original study and for being very responsive in answering any questions we had in trying to replicate the original study as precisely as possible. Our study did not use the original code, however, because Tim Brady pointed out that it would not work with modern web browsers, and it was not shared with us.

5 Please note that most current operating systems automatically apply a scaling factor for high-dpi displays and that users can adjust the scaling factor in modern browsers. Thus, the real number of pixels in such cases is likely higher, which then enables individuals to see the stimulus display at a convenient physical size. This applies to all pixel values given in this paper.
